# The law of non-usage attrition in a technology-based behavioral intervention for black adults with poor cardiovascular health

**DOI:** 10.1371/journal.pdig.0000119

**Published:** 2022-10-25

**Authors:** Muhammed Y. Idris, Mohamed Mubasher, Ernest Alema-Mensah, Christopher Awad, Kofi Vordzorgbe, Elizabeth Ofili, Arshed Ali Quyyumi, Priscilla Pemu

**Affiliations:** 1 Department of Medicine, Morehouse School of Medicine, Atlanta, Georgia, United States of America; 2 Clinical Research Center, Morehouse School of Medicine, Atlanta, Georgia, United States of America; 3 Community Health and Preventive Medicine, Morehouse School of Medicine, Atlanta, Georgia, United States of America; 4 Department of Medicine, Emory University School of Medicine, Atlanta, Georgia, United States of America; 5 Emory Clinical Cardiovascular Research Institute, Emory University, Atlanta, Georgia, United States of America; Flinders University, AUSTRALIA

## Abstract

Digital health innovations, such as telehealth and remote monitoring, have shown promise in addressing patient barriers to accessing evidence-based programs and providing a scalable path for tailored behavioral interventions that support self-management skills, knowledge acquisition and promotion of relevant behavioral change. However, significant attrition continues to plague internet-based studies, a result we believe can be attributed to characteristics of the intervention, or individual user characteristics. In this paper, we provide the first analysis of determinants of non usage attrition in a randomized control trial of a technology-based intervention for improving self-management behaviors among Black adults who face increased cardiovascular risk factors. We introduce a different way to measure nonusage attrition that considers usage over a specific period of time and estimate a cox proportional hazards model of the impact of intervention factors and participant demographics on the risk of a nonusage event. Our results indicated that not having a coach (compared to having a coach) decreases the risk of becoming an inactive user by 36% (HR = .63, P = 0.04). We also found that several demographic factors can influence Non-usage attrition: The risk of nonusage attrition amongst those who completed some college or technical school (HR = 2.91, P = 0.04) or graduated college (HR = 2.98, P = 0.047) is significantly higher when compared to participants who did not graduate high school. Finally, we found that the risk of nonsage attrition among participants with poor cardiovascular from “at-risk” neighborhoods with higher morbidity and mortality rates related to CVD is significantly higher when compared to participants from “resilient” neighborhoods (HR = 1.99, P = 0.03). Our results underscore the importance of understanding challenges to the use of mhealth technologies for cardiovascular health in underserved communities. Addressing these unique barriers is essential, because a lack of diffusion of digital health innovations exacerbates health disparities.

## Author summary

Mobile-health (mHealth) technologies continue to serve immensely valuable roles in current healthcare systems by bridging several accessibility gaps and facilitating patient-provider interactions. However, and ironically, they also present several accessibility gaps due to design features and/or individual characteristics. In this study, we explore the effects of having an adjunct health coach with a behavior-modification mHealth app, Health360x (H360x) in promoting user-interface interaction in a population of African American patients with significant cardiovascular (CV) risk factors. While we saw an improvement in CV risk factors, we also identified an interesting attrition in app usage in patients assigned a health coach; and observed similar effects in those with higher socioeconomic status. These findings may be due to a higher (baseline or acquired) self-efficacy in these patients, and suggests that longitudinal assessments of app engagement in this population should be approached with careful consideration of attrition.

## Introduction

Cardiovascular disease (CVD) is the leading cause of death in the United States and disproportionately impacts racial and ethnic minorities, particularly Black/African American patients, who have a CVD risk factor-adjusted death rate 30% higher than their non-Hispanic white counterparts [1]. African American patients face disparities in incidence of CVD-related events, including stroke, myocardial infarction, heart failure, and acute coronary syndrome, leading to significant functional impairment and mortality [2]. Risk factors associated with CVD-related disparities in Black communities include higher prevalence of hypertension, diabetes, and obesity [3]. Disparities in health behaviors, including physical activity, smoking, greater than moderate drinking, unhealthy diets, and psychological distress, are associated with these risk factors [3]. Lifestyle changes (i.e., physical activity, diet, adherence to medication, and smoking) and adherence to cardiac rehabilitation have a significant impact on the risk of future CVD events, but even these are hindered by marked racial and ethnic disparities in participation in the use of cardiac rehabilitation among eligible patients in the U.S. [4].

Previous research has shown that tailored behavioral interventions can contribute to the improvement of key lifestyle factors that contribute to ideal cardiovascular health among Black/African American patients [5, 6]. However, targeting and tailoring lifestyle interventions for preventing and managing CVD can be labor intensive, cost prohibitive, and inaccessible to underserved patient populations. Recent technological innovations, including telehealth and remote monitoring, have shown promise in addressing patient barriers to accessing evidence-based programs and providing a scalable path for tailored behavioral interventions that support self-management skills, knowledge acquisition and promotion of relevant behavioral change [7]. However, significant attrition continues to plague internet-based studies, a result that can be attributed to characteristics of the intervention, or individual user characteristics.

Intervention factors include familiarity and adaptability to remotely connected smart devices, usability problems or complexity, ease of discontinuance, not observing early advantage to its use as well as the existence of a trusted coach [5, 8]. Individual characteristics that contribute to attrition include demographic factors like socioeconomic status, education, less contact with change agents and poor access to technology [25]. This is even more noticeable among minority and underserved populations, who experience challenges accessing reliable health sources on the web, navigating technology, and accessing sources in their respective language and appropriate reading levels in order to make informed health decisions [9]. While others have systematically examined attrition in controlled trials [10] and real-world applications [11] of behavioral intervention technologies (BITs), there are currently no studies that consider the role of race/ethnicity on attrition rates, engagement measures, and associated variables. Moreover, it is important to distinguish between dropout attrition (i.e., participants are lost to follow-up) and nonusage attrition (i.e., participation in the study, but are not engaging with BITs). As Eysenbach (2005) notes, “the loss-to-follow-up attrition curve usually follows the nonusage attrition curve because a high proportion of loss to follow-up is a result of nonusage (“losing interest” is the underlying variable which explains both curves)” [12].

In this paper, we provide the first analysis of the determinants of nonusage attrition in a randomized control trial of a technology-based intervention for improving self-management behaviors among Black adults with multiple cardiovascular risk factors. In what follows we introduce the Morehouse-Emory Cardiovascular (MECA) Study, which investigated multi-level exposures that explain intraracial and intraethnic differences in cardiovascular health outcomes. We then describe our methodology for measuring nonusage attrition from user logs exported from Health360x, a coach-assisted consumer health information technology (CHIT) designed to support chronic illness care through behavior change. We present attrition curves of nonusage over the 6-month intervention overall and as a function of the intervention arm. Our hypothesis is that attrition is influenced by a combination of intervention and individual characteristics.

## Methods

### Morehouse-Emory Cardiovascular (MECA) clinical intervention

The data used to assess the determinants of attrition with a behavioral intervention technology among Black adults with poor cardiovascular health was taken from a clinical intervention as part of the Morehouse-Emory Cardiovascular (MECA) Center for Health Equity Study [13]. The parent MECA study was primarily concerned with exploring intraethnic and intraracial differences in cardiovascular health outcomes. The Institutional Review Boards of both Morehouse School of Medicine and Emory University School of Medicine approved this research under an IRB Authorization Agreements #784479-2 and #00083584, respectively. Consent was obtained orally and documented by research coordinators in participant surveys. Written consent could not be obtained because participants were enrolled via telephone.

Poor cardiovascular health was defined by calculating a participant’s Life’s Simple 7 score, seven CVD risk factors as defined by the American Heart Association (AHA): smoking status, physical activity, weight, diet, blood glucose, cholesterol, and blood pressure [14]. Research has shown that patients with ideal scores in at least five risk factors over 4-7 years experienced 78% and 88% reductions in all-cause mortality and mortality associated with CVD, respectively [15]. Participant risk factors were measured during baseline visits in the parent MECA study. LS7 scores were calculated using guidelines set forth by the AHA, with score ranges between 0–14, giving 2 points for Ideal, 1 point for Intermediate, and 0 points for Poor components [16]. All participants in the parent MECA study with low LS7 scores (i.e., an LS7 composite score of 8 or lower) who agreed to be contacted for the clinical intervention study were called and consented by the research staff. Additional inclusion and exclusion criteria can be found in [5].

The goal of the 6-month MECA clinical intervention (the focus of this manuscript) was to influence lifestyle changes that are associated with ideal cardiovascular health, while addressing barriers to technology-enabled self-management of CVD through the use of trained health coaches. All participants received a web- and mobile-based behavioral intervention technology, Health360x, and were randomized into two groups; one group receiving an adjunct health coach (high tech-high touch) and the other using only Health360x (high tech). All participants were trained to use Health360x to monitor and manage cardiovascular risk factors including, blood sugar, hypertension, BMI, and total cholesterol, while improving their lifestyles in terms of diet, tobacco use, and physical activity. Before being randomized, all participants were trained on Health360x and assessed on their knowledge and ability to use Health360x. In a previous study, we’ve shown the benefit of engagement with Health360x and the role that health coaches can play in empowering participants to develop self-management skills that were related to improvements in clinical outcomes including reductions in blood pressure and blood glucose levels [8]. We tested the hypothesis that an intervention that combines technology with a trusted partner (high tech-high touch) will be more effective in engaging patients, increasing attainment of self-management goals, and ultimately improve cardiovascular health. Our results from this multi-faceted technology-enabled and coach-assisted clinical intervention indicated that our intervention can lead to improvements in cardiovascular health (as measured by LS7) corresponding to a 7% lifetime reduction of CVD incidence CV, and that health coaches can help improve overall LS7 in participants living in at-risk neighborhoods [5].

### Health360x: Coach-assisted platform for chronic illness care

**Fig 1:**
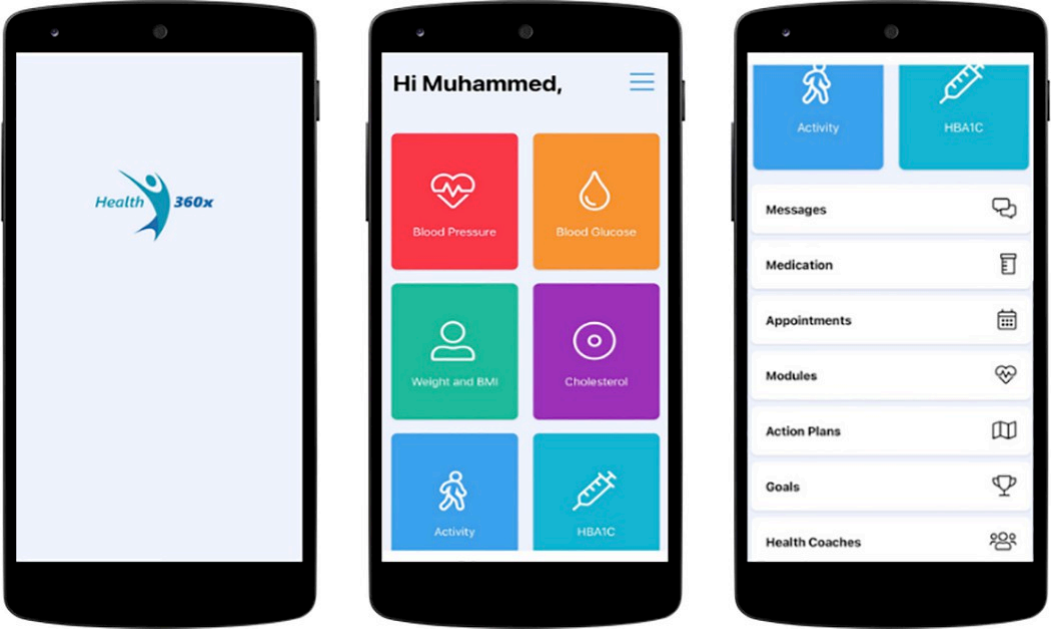
Screenshots of Health360x.

Health360x (Accuhealth, Atlanta, USA) is a proprietary application developed by researchers at Morehouse School of Medicine to support self-efficacy for self-management of chronic illnesses. Fig 1 provides screenshots of Health360x. This patented technology provides a variety of functionality ranging from built-in coaching to help overcome digital and health literacy barriers and promote self-efficacy; social network to promote motivation and community; illness-specific content to empower users with knowledge to improve their health condition; and a tracking system to record blood pressure, BMI, physical activity, and self-management through manual input or remote monitoring. All consented participants were trained on Health360x and then completed a brief usability test which included task scenarios around logging on, uploading and viewing data, setting and tracking behavioral goals, finding and completing study-related questionnaires, sending emails to the study team, posting to an online community forum, and accessing educational content. Participants also completed a brief test of their ability to use the core functionality of Health360x after training. All participants were had access to 24/7 technical support, but participants in the high tech-high touch group also had access to coaches to provide additional support with technical assistance.

#### Underlying study design considerations: Treatment assignment

The intervention is designed to compare two approaches to engagement and in turn behavior change: technology alone (high tech only) versus technology coupled with a health coach (high tech-high touch). Only participants who successfully completed a usability test were randomly assigned to an intervention arm using a time-varying-permuted block size randomization algorithm with stratification based on gender and neighborhood/community prevalence of cardiovascular morbidity and mortality. Participants in the high tech-high touch group were randomly assigned to work with a coach in person or by phone every week for the 1st month, then every two 2 weeks for 8 weeks, and finally monthly for 3 months to develop and adapt a personalized action plan to monitor and manage CVD risk and improve lifestyles.

#### Covariates (Mediators, effects Modifiers analyses variables)

**Participant demographics**. Participant demographics including age, gender, marital status, income, education, and employment were collected during patient visits at Emory University Hospital and Morehouse School of Medicine during the baseline MECA clinical study. To determine whether a patient lived in a neighborhood/community that enhances or reduces CVD risk, participant addresses were mapped to census tracts. Census-tract level data on CVD-related related deaths and emergency department admissions for black adults between 35 and 64 from 2010 to 2014 was used to classify communities as ‘at risk’ for or ‘resilient’ against cardiovascular morbidity and mortality [17]. Finally, we included a participant’s baseline Life’s Simple 7 score as an independent variable.

**Primary analysis outcome: Nonusage attrition**. This was defined as a single or multiple events, thus measuring the intensity of nonusage. To measure nonusage attrition, we exported usage logs of time-stamped events for all participants in the study from Health360x. We then filtered usage logs to only include events that are relevant to the primary function of the app, including logging weight, blood pressure, blood glucose, medication, cholesterol, and appointments as well as creating/modifying action plans, sending messages, and filling out study specific surveys. We measured nonusage attrition by considering both the duration and intensity of usage. We could not find any literature that proposed a standard approach for defining a threshold for meaningful usage, so we elected to use the first quartile of the number of events by users as a cutoff. A participant was coded as inactive and a nonusage event was logged if they did not log a minimum of 9 relevant events in the last 24 hours. It is possible that a participant does not experience any nonusage events if they never reach the threshold of 9 events (i.e., a user logs in and somehow interacts with the app, but doesn’t perform any of the previously mentioned list of events that we would record as a ‘usage event’). It is also possible that participants can oscillate between being active and inactive users which is a natural feature of engagement with software applications. As such, it is possible to also possible observe multiple nonusage events for a given participant even if they may indeed be engaging with the app.

### Research ethics

All procedures and protocols were conducted in accordance with the Emory University and Morehouse School of Medicine Institutional Review Boards (IRBs). Briefly, participants enrolled in the MECA study were consented in by phone and expressed adequate understanding of the study. Researchers were also adequately trained in educating participants and ensuring proper handling and security of patient data throughout the study. The digital data used for analysis in this article was highly secured, with security measures monitored annually by a an advisory/oversight committee to address any potential adverse events. Throughout this study, data access was restricted only to the appropriate investigators as required by IRB guidelines.

The study was also designed to ensure an adequate representation of different minority groups within the population in question. Patient demographics were estimated from statistics reported by the Morehouse Choice Accountability Care Organization; and we anticipated a 60:40 female to male ratio, with close to 65% of participants belonging to a minority group. While several demographic factors could potentially impact our outcomes, we did not discover any notable differences in our population in some of these variables (Table 1). However, we recognize that other factors like digital literacy and self-efficacy could strongly influence attitudes towards mHealth apps. We sought to address these by training our participants in the use of the app to a minimum competency level and randomly assigned them to having a coach or not. By design, our outcome measures analyzed the frequency of interaction with the app, and less on the proficiency of the user. Additionally, randomly assigning proficient and less proficient users to coaches would aim at reducing the effect of self-efficacy while highlighting the role of having a coach on usage attrition.

**Table 1:** Demographics of participants.

	N (%)	Mean (SD)	P-Value
***Intervention Arm***			
Coach	61 (46%)	-	-
No Coach	71 (54%)	-	-
***Age***	132 (100%)	55.20 (8.83)	0.60
***Gender***			
Female	93 (71%)	-	-
Male	39 (30%)	0.77	
***Baseline Life’s Simple 7 ≥ 6***	132 (100%)	6.23 (1.47)	0.14
***Marital Status***			
In Relationship	29 (22%)	-	-
Divorced	47 (36%)	-	0.35
Widowed	10 (8%)	-	0.97
Separated	8 (6%)	-	0.52
Never Married	38 (29%)	-	0.96
***Income***			
Less than $15,000	42 (32%)	-	0.94
15,000 *to less than* 35,000	34 (26%)	-	0.79
35,000 *to less than* 50,000	19 (14%)	-	0.44
50,000 *to less than* 75,000	17 (13%)	-	0.02
More than $75,000	16 (12%)	-	-
***Education***			
Grade 9-11 (Some high school)	12 (9%)	-	-
Grade 12 or GED (High school graduate)	25 (19%)	-	0.11
College 1 year to 3 years (Some college or technical school)	46 (35%)	-	0.26
College 4 years or more (College graduate)	49 (37%)	-	0.33
***Employment***			
Employed, full or part-time	58 (44%)	-	-
Employed, but temporarily away from my regular job	2 (2%)	-	0.98
Unemployed, looking for work	11 (8%)	-	0.07
Unemployed, not looking for work	46 (35%)	-	0.26
Retired from my usual occupation but working for pay	15 (11%)	-	0.15
***Neighborhood CVD Risk (Census Tract)***			
Resilient	20 (15%)	-	-
At Risk	23 (17%)	-	0.74
None of the Above	89 (67%)	-	0.74

While the study focuses on a specific patient population (i.e. elderly African-Americans with CVD risk factors), we hope that the insights gained would be useful in understanding the efficacy of interventional and therapeutic designs in achieving their goals based on demographic features of the target population. mHealth apps provide a unique challenge in the domain of patient-provider interactions because they serve to automate one or more aspects of this relationship. Understanding the role of demographic factors on the efficacy of these apps-based interventions, as endeavored by this study, is essential in ensuring an improved and worthwhile experience.

### Statistical considerations

#### Statistical power

Using nonusage attrition as a single event we estimated statistical power using variable scenarios of hazard ratios of events between the two high-tech and high tech-high touch groups.

#### Statistical analysis

**Participant demographics**. The participants’ characteristics, including demographics, baseline, and 6-month changes in CVD risk variables were summarized and compared by randomization groups (high tech vs. high tech-high touch). Binary/categorical variables like sex at birth, education, and the presence of diabetes were summarized using frequencies and percentages. Continuous variables like age, BMI, and blood glucose were summarized by means and standard deviations. We univariately tested balance of patients characteristics and predictive covariates between the two randomization groups using univariate logistics function with the likelihood of being on one of the randomization groups as an outcome vis-à-vis each of the potential predictors of the outcome measures, to determine any association with patient’s baseline characteristics. Statistically insignificant p-values indicate that the patient characteristic (e.g., age) is not correlated with likelihood of assignment to randomization group suggesting comparability between groups.

**Nonusage attrition**. As suggested by [12], formal analysis of attrition can be done using survival analysis to assess the association between predictor variables and the time until a nonusage attrition event occurs. We plot attrition curves and test the null hypothesis of no difference in survival between stratified groups using the log rank test. The semi-parametric Cox proportional-hazards regression model is the workhorse of survival modeling and is widely used in biomedical research to investigate the influence of multiple independent variables on time-to-event of interest [18]. In this simplest form, the Cox model is often used to model single events. We also tested for intensity of nousage attrition, since it is likely that participants will oscillate between being active and inactive users and thus the event of interest (nonusage attrition), is likely to occur more than once in a participant, a phenomenon known as intensity of or recurrent events. Several methods have been proposed to analyze adapt the Cox model for multiple events and differ in their assumptions about the dependency of events. We elected to use a frailty model because it assumes events are independent except for subject-specific random effects which account for unmeasured heterogeneity that cannot be described by predictors in the model alone [19]. Like linear mixed-effects models, regression estimates in the Cox regression model with random effects are subject-specific and should be interpreted as such. Sample size permitting we explored interactions between treatment assignment (i.e., Coach, No Coach) and a participant’s baseline Life’s Simple 7.

## Results

**Fig 2:**
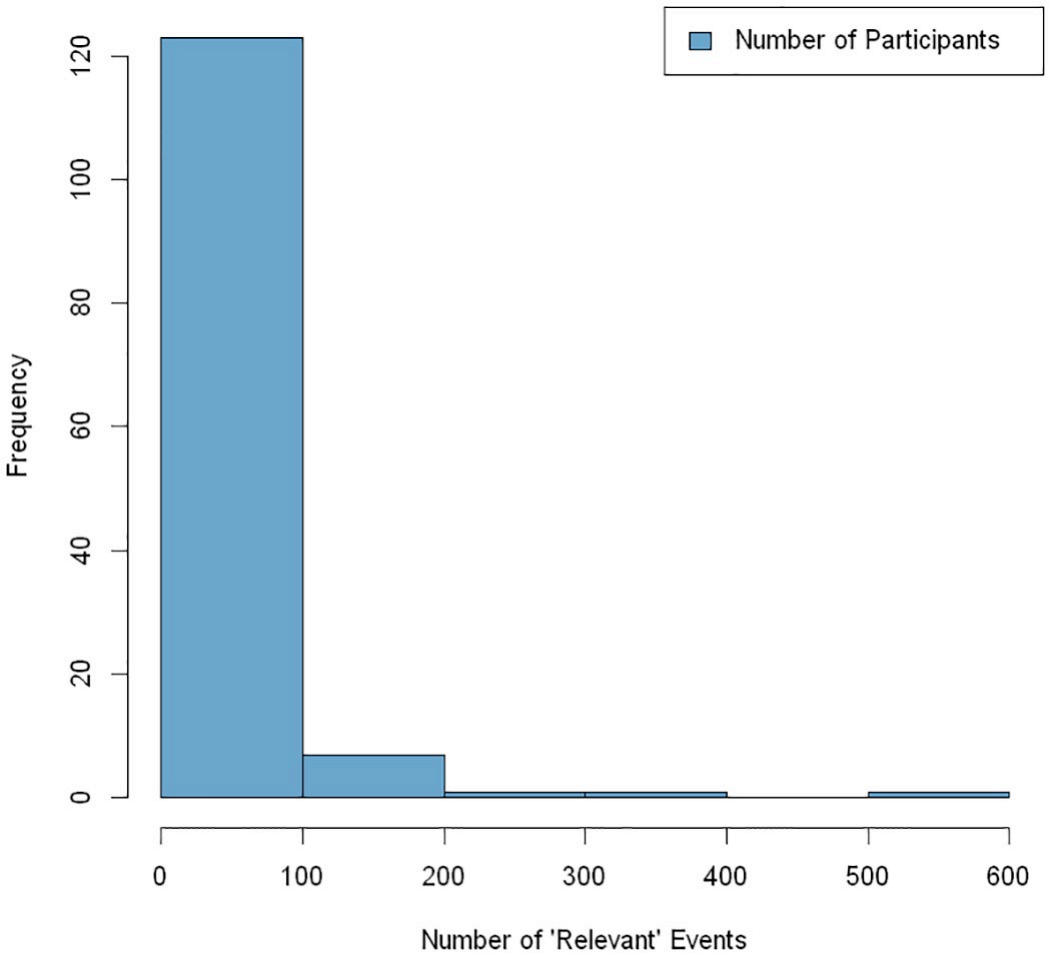
Histogram of user events relevant to the primary function of Health360x.

Table 1 presents demographics in our overall sample of 146 participants in the MECA clinical intervention study. Fig 2 represents the frequency of user events or actions relevant to the primary function of Health360x (i.e., changing passwords vs. logging blood pressure). The number of relevant user actions over the course of the study ranged from 2 to 522, with an average of 38.27 (SD = 62.31) events per MECA participant.

**Fig 3:**
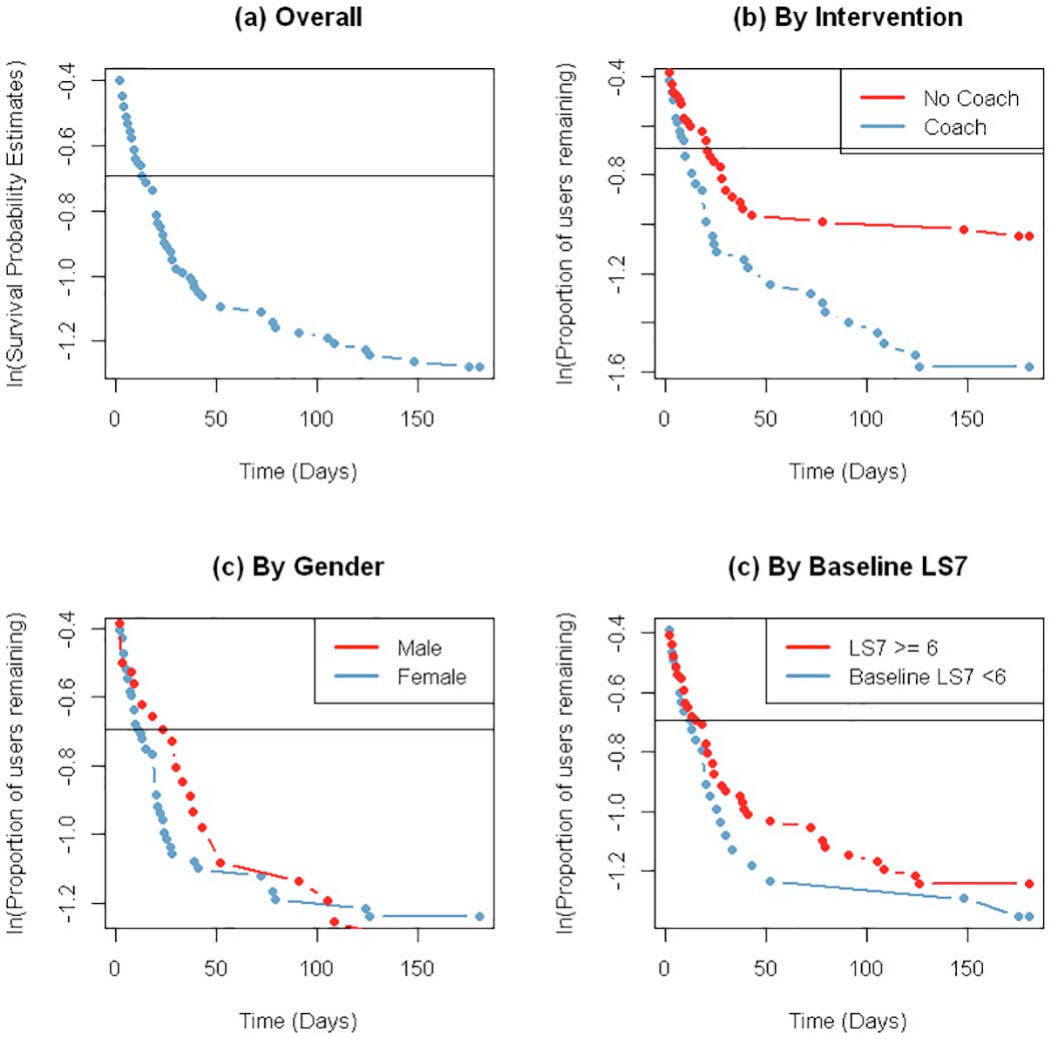
Logarithmic nonusage attrition curves in the MECA clinical intervention.

Fig 3 presents nonusage attrition curves of participants in the MECA Clinical Intervention, where the y axis is the log of the proportion of users remaining who have not experienced a nonusage event. Fig 3A indicates that the median time to nonusage attrition when looking at the overall sample is 13 days. Fig 3B stratifies participants by intervention group. The median survival time for participants in the high tech-high touch group (i.e., Coach) is 9.5 days, while the median survival time in the high tech group (i.e., No Coach) is 20.5 days. Non-intersecting attrition curves when stratifying by intervention group indicate statistically significant differences in survival between strata. We can therefore conclude that there is a significant difference in nonusage attrition between the intervention groups. Fig 3C and 3D compare attrition curves by gender and baseline Life’s Simple 7 scores, respectively. The median survival time for male participants was 23 days and female participants 11 days. The median survival time for participants with baseline LS7 scores greater than or equal to 6 was 15 days, and those with LS7 scores smaller than 6 was 12 days. Results from log rank tests indicate that there is no statistically significant differences in attrition based on gender (P = 0.99) or baseline LS7 (P = 0.60), but there is a significant difference in attrition as a function of (P = 0.04).

Table 2 presents estimates from a Cox proportional hazard model of participant nonusage attrition conditional on unmeasured heterogeneity and intervention and participant characteristics. Model estimates suggest that compared to the Coaching intervention group, there is a 36% decrease in the risk of a participants in No Coach intervention group moving from being active to inactive users (HR = 0.64, P = 0.04). Results also suggest that the risk of nonusage attrition among participants who completed some college or technical school (HR = 2.91, P = 0.04) or graduated college (HR = 2.98, P = 0.047) is 3 times higher than those who did not graduate high school. Finally, we find that the risk of nonusage attrition among participants with poor CVD health from at risk neighborhoods is approximately two times the risk of participants from resilient neighborhoods (HR = 1.99, P = .03). We did not find evidence that marital status, income, or employment status are significant predictors of nonusage attrition in this population. We tested the proportional hazards assumption for the Cox regression model fit by checking for time dependency using the Schoenfeld residuals against the transformed time for reach predictor in our model. No predictors were statistically significant indicating that our model meets the proportional hazards assumption.

**Table 2:** Estimates of the Cox proportional hazard model of nonusage attrition.

	*β* (se)	*P-Value*	HR (95% CI for HR)
No Coach	-0.46 (0.22)	0.041	0.63 (0.41-0.98)
Age	0.01 (0.02)	0.498	1.01 (0.98-1.04)
Gender (Ref = Female)	0.17 (0.22)	0.448	1.18 (0.77-1.81)
*Baseline Life’s Simple 7 ≥ 6*	-0.07 (0.08)	0.395	0.94 (0.80-1.08)
***Marital Status (Ref = Married)***			
Divorced	-0.04 (0.24)	0.867	0.96 (0.60-1.54)
Widowed	0.59 (0.38)	0.118	1.81 (0.86-3.81)
Separated	0.07 (0.53)	0.895	1.07 (0.38-3.00)
Never Married	0.32 (0.25)	0.236	1.38 (0.81-2.35)
***Income (Ref = More than $75,000)***			
Less than $15,000	-0.13 (0.36)	0.713	0.88 (0.44-1.77)
15,000 *to less than* 35,000	-0.43 (0.34)	0.193	0.65 (0.33-1.25)
35,000 *to less than* 50,000	-0.35 (0.35)	0.322	0.70 (0.35-1.41)
50,000 *to less than* 75,000	-0.31 (0.38)	0.414	0.74 (0.35-1.53)
***Education (Ref = Some high school)***			
Grade 12 or GED (High school graduate)	0.48 (0.55)	0.382	1.62 (0.55-4.77)
College 1 year to 3 years (Some college or technical school)	1.16 (0.52)	0.027	3.19 (1.14-8.92)
College 4 years or more (College graduate)	1.16 (0.54)	0.032	3.18 (1.10-9.16)
***Employment (Ref = Employed, full or part time)***			
Employed, but temporarily away from my regular job	-0.13 (1.08)	0.901	0.87 (0.11-7.25)
Unemployed, looking for work	0.36 (0.41)	0.388	1.43 (0.64-3.22)
Unemployed, not looking for work	0.04 (0.31)	0.905	1.03 (0.56-1.92)
Retired from my usual occupation but working for pay	0.08 (0.38)	0.839	1.08 (0.51-2.27)
***Neighborhood CVD Risk (Census Tract)***			
At Risk	0.69 (0.31)	0.026	2.00 (1.03-3.87)
None of the Above	-0.02 (0.26)	0.926	0.98 (0.60-1.63)
*N* = 188; *Number of Nonusage Attrition Events* = 136			

## Discussion

### Principal findings

In this study we have presented a first-of-its-kind analysis of nonusage attrition in a technology-based behavioral intervention for Black/African American patients with poor cardiovascular health. Despite a high retention rate of over 90% in the study, we found that having a coach was likely to increase the risk of nonusage. We also found that several demographic factors can influence Non-usage attrition: Higher educational attainment was associated with greater rate of nonusage. This finding suggests, counterintuitively, that education may not play a protective role in promoting engagement with behavioral intervention technologies within Black communities. Finally, we found that the risk of nonusage attrition among participants with poor cardiovascular from “at-risk” neighborhoods with higher morbidity and mortality rates related to CVD is significantly higher when compared to participants from “resilient” neighborhoods. We also explored interaction effects, specifically the interaction treatment group by baseline Life’s Simple 7 dichotomized by the median of participant scores and the result did not show any significant difference in nonusage attrition. There was some imbalance between treatment groups based on income (specifically participants whose income was between $50,000 and $75,000, but income was not a significant predictor in our Cox regression model.

Some key considerations in mHealth app usage are prior digital literacy, technological self-efficacy, and perceived utility especially on the part of the participants. While we ensured a minimum competency level in navigating the app, we recognize that this did not guarantee a lack of an effect on app usage. We sought to minimize this effect by analyzing the frequency of interaction with the app regardless of the proficiency of the user so long as they appropriately completed a required basic function. The app was also designed with careful consideration of accessibility requirements, which include the ease of use and the need for fewer steps to complete an action. While we did not measure the effects of accessibility provisions on usage, a value of this study is to provide a useful analytical tool for future research of this relationship. Indeed, an intriguing question would be the role (or lack of a role) of coaches on surmounting usability challenges and encouraging use even in apps that fall short of adequate accessibility provisions.

Another potential contributor was patient fatigue, both in using the app and in participating in the general study. This was a unique challenge in the sense that the effect of this fatigue could more directly affect our outcome regardless of proficiency or self-efficacy. We sought to minimize this issue using randomization of participants into coaching groups, and also gradually reduce and taper the interactions with coaches still ensuring availability of assistance if needed. Future studies may further illuminate this effect using questionnaires at throughout and at the end of the study to assess willingness to perform key basic tenets of the study and correlating them with attrition and proficiency rats. On the part of this study, we noted a non-usage effect only after a minimum number of events (9) was met in 24 hours. This minimum represented the first quartile of events by users in our cohort, since we did not find any reliable usage thresholds in the literature. While this measure may still provide a technical demand for some patients who may be less comfortable or adept at using mobile devices, we believed it represented an adequate minimum usage for the designed purpose of the H360x app. However, it may be useful in the future to further explore these minimum thresholds and correlate them with app usage and perceptions, especially in populations susceptible to these effects.

Finally, the perceived utility of an app is a key feature in a patient’s usage of the app. While part the role of the coach was to foster this perceived utility, we recognize that this effect is strongly individual and may be difficult to assess given the varying degrees of digital literacy and efficacy. Interestingly, we noted an increase in attrition in individuals with a relatively higher education. It is conceivable that a higher education level would be accompanied by an increased willingness and ability to engage the app [20]. Conversely, the effect observed in this study may be due to a perceived lack of utility by patients who may feel less of a need to rely on this app as an adjunct tool in their healthcare. As with patient fatigue/willingness to use the app, periodic targeted questionnaires throughout the study may help to shed light on these effects and correlate them with usage attrition.

## Limitations

Several limitations of this study are worth considering. First, the technology infrastructure underlying Health360x went through some significant changes during the intervention. While the features and functionality of the platform remained the same, the backend was revamped which led to participants facing some technical issues during development (e.g., unable to sign-in to profiles) and differences in how usage data was defined and collected. The latter issue around usage data limited our measurement of user engagement with Health360x to variables that captured when and how long participants used Health360x. Second, it is important to note the quality of coaches varies and as such may have a differential effect on nonusage attrition. While we tried to emphasize uniform training for coaches, it is likely that their personal and cultural experiences influence their interactions with participants. Our results are also likely sensitive to the threshold of 9 events that are relevant to the primary function of Health360x and a 24 hour time period. More work is needed to create a systematic approach for understanding and modeling model dose-response in app based digital health interventions. Another limitation is that data on digital health literacy was not collected from participants, which may indicate omitted variable bias.

## Conclusion

Non-usage attrition is an ongoing concern because it can significantly impair the efficacy of behavioral intervention technologies (BITs) irrespective of its design benefits. Key contributors to nonusage attrition can be broadly described in terms of individual (user) characteristics and intervention factors [21, 25]. The primary goal of this study was to characterize determinants of usage attrition in a behavioral intervention technology, Health 360x, within a population of Black patients with poor cardiovascular health. Using individual level characteristics like education and other socioeconomic factors, we found that several demographic factors influence a participant’s risk of lapsing into nonusage attrition across several weeks, including education-level. A possible explanation that we will assess in future studies is that who receive more education may face a greater psychological burden of being the ones who made it and with that an added responsibility to provide. These observations illustrate a need for a nuanced understanding of how technology-enabled interventions disparately impact underserved communities and can lead to greater disparities.

We did not find evidence that gender, age, baseline Life’s Simple 7, marital status, income and unemployment influenced nonusage attrition. Other studies have found mixed evidence of how these factors influence nonusage attrition. Analysis of nonusage attrition in a randomized control trial of a web-based smoking cessation intervention found that relatively young, highly educated, and unmarried participants are at greater risk of nonusage attrition compared to older participants from high socio-economic backgrounds as well as older female participants from low SES backgrounds [22]. In an observational study of a physical activity program in Australia, researchers found a lower mean time to attrition among participants who were men and inconsistent results with respect to age [23, 24]. Comparing outcomes in a 12 week vs 52 week web-based weight loss intervention, Neve et al. found that age is associated with nonusage attrition in the short tem, but it’s influence dissipates over time [25]. We could find no studies that explore the impact of race and ethnicty on nonusage attrition.

Additionally, we tested the potential benefits of having an adjunct health coach to determine if this multi-faceted intervention was associated with nonusage attrition. Previous research has shown the positive impact of having a coach on short-term behavior modification [26]. The findings of this study suggest that the observed long-term benefits of a behavior-change technology with a coach may not be as closely related to constant BIT use, but rather on the coach-patient relationship and the participants self-efficacy for self-management [8, 27]. Indeed, it is possible that the perceived benefits of having a coach may reduce a patient’s diligent use of the BIT, as the coach assumes a more direct role in influencing behavioral change. Furthermore, it is important to consider individual differences in motivation style, as some patients who prefer a less ‘hands-on’ approach may find a coach-based intervention cumbersome. Further work should be done to create new measures to capture the multidimensionality of mHealth BIT usage, explore efficacy of BITs as a function of usage, and investigate the impact of social determinants on nonusage attrition, especially in the presence of adjunctive interventions like health coaches and peer navigators that we have shown affect usage. Future studies will explore interactions between treatment group, demographic variables, and social determinants.
